# Manganese: The overlooked contaminant in the world largest mine tailings dam collapse

**DOI:** 10.1016/j.envint.2020.106284

**Published:** 2020-11-29

**Authors:** Hermano M. Queiroz, Samantha C. Ying, Macon Abernathy, Diego Barcellos, Fabricio A. Gabriel, Xosé L. Otero, Gabriel N. Nóbrega, Angelo F. Bernardino, Tiago O. Ferreira

**Affiliations:** a Luiz de Queiroz College of Agriculture, University of São Paulo (ESALQ-USP), Av. Pádua Dias 11, CEP 13418-900, Piracicaba, São Paulo, Brazil; b Environmental Toxicology Graduate Program, University of California, Riverside, CA 92521, United States; c Department of Environmental Sciences, University of California, Riverside, CA, 92521, United States; d Grupo de Ecologia Bentônica, Departamento de Oceanografia, Universidade Federal do Espírito Santo, Vitória, Espírito Santo 29075-910, Brazil; e Department of Edaphology and Agricultural Chemistry - CRETUS, Faculty of Biology, Universidade de Santiago de Compostela, Campus Sur, 15782, Santiago de Compostela, Spain; f Graduate Program in Earth Sciences (Geochemistry), Department of Geochemistry, Federal Fluminense University, Niterói, Brazil

**Keywords:** Estuarine soils, Manganese contamination, Iron oxides, Redox processes, Toxicity

## Abstract

Manganese (Mn) is an abundant element in terrestrial and coastal ecosystems and an essential micronutrient in the metabolic processes of plants and animals. Mn is generally not considered a potentially toxic element due to its low content in both soil and water. However, in coastal ecosystems, the Mn dynamic (commonly associated with the Fe cycle) is mostly controlled by redox processes. Here, we assessed the potential contamination of the Rio Doce estuary (SE Brazil) by Mn after the world’s largest mine tailings dam collapse, potentially resulting in chronic exposure to local wildlife and humans. Estuarine soils, water, and fish were collected and analyzed seven days after the arrival of the tailings in 2015 and again two years after the dam collapse in 2017. Using a suite of solid-phase analyses including X-ray absorption spectroscopy and sequential extractions, our results indicated that a large quantity of Mn^II^ arrived in the estuary in 2015 bound to Fe oxyhydroxides. Over time, dissolved Mn and Fe were released from soils when Fe^III^ oxyhydroxides underwent reductive dissolution. Due to seasonal redox oscillations, both Fe and Mn were then re-oxidized to Fe^III^, Mn^III^, and Mn^IV^ and re-precipitated as poorly crystalline Fe oxyhydroxides and poorly crystalline Mn oxides. In 2017, redox conditions (Eh: −47 ± 83 mV; pH: 6.7 ± 0.5) favorable to both Fe and Mn reduction led to an increase (~880%) of dissolved Mn (average for 2015: 66 ± 130 μg L^−1^; 2017: 582 ± 626 μg L^−1^) in water and a decrease (~75%, 2015: 547 ± 498 mg kg^−1^; 2017: 135 ± 80 mg kg^−1^) in the total Mn content in soils. The crystalline Fe oxyhydroxides content significantly decreased while the fraction of poorly ordered Fe oxides increased in the soils limiting the role of Fe in Mn retention. The high concentration of dissolved Mn found within the estuary two years after the arrival of mine tailings indicates a possible chronic contamination scenario, which is supported by the high levels of Mn in two species of fish living in the estuary. Our work suggests a high risk to estuarine biota and human health due to the rapid Fe and Mn biogeochemical dynamic within the impacted estuary.

## 1. Introduction

Manganese (Mn) is a widely distributed element in terrestrial and coastal ecosystems but usually occurs as trace amounts in most organisms (Levy and Nassetta, 2003; Pinsino et al., 2012). It is found in rocks (e.g., Mn content in Basalt: 1300 mg kg^−1^; Gneiss: 600 mg kg^−1^; Limestone: 550 mg kg^−1^; Graham et al., 1988), associated with different primary soil minerals (Mn content in amphiboles: 400–7000 mg kg^−1^; olivines: 100–6500 mg kg^−1^; pyroxenes: 600–8000 mg kg^−1^; Graham et al., 1988), and dissolved in natural waters (e.g., dissolved Mn in oceanic water ranges from 0.2 to 5.0 nmol kg^−1^; Graham et al., 1988), exhibiting unique redox dynamics (as Mn^II^, Mn^III^, and Mn^IV^) with Mn^IV^ being the most abundant form found in minerals (Burdige, 1993; Fischel et al., 2015).

For all living organisms, Mn is required in small amounts playing important roles in the maintenance of different biological functions and life (Arndt et al., 2014; Baly, 1989; Pinsino et al., 2012). For instance, in plants, Mn plays a key role in enzymatic activities and cell division (Broadley et al., 2012), while in humans and animals Mn acts as a protein transporter, is involved in neurological functions, and can also affect enzymatic activities (Fitsanakis et al., 2010). The required trace amounts of Mn considered beneficial to life are variable. In plants, such as soybean and corn, the critical quantity of Mn to reach toxic levels is 200 mg Mn kg^−1^ leaf dry weight (El-Jaoual and Cox, 1998). For humans, the World Health Organization, (WHO, 2011) set adequate intake levels for Mn at 2–3 mg day^−1^.

The many physiological roles of Mn have often masked the perception of its potential toxicity, hence studies are rare that focus on the severe toxic effects produced by this element in different environments such as water, soil, and air (Finkelstein and Jerrett, 2007; Huang et al., 2016; Li et al., 2007). Thus, Mn often remains unnoticed as a contaminant due to its role as a micronutrient for plants and animals and to its ubiquity in the environment (Pinsino et al., 2012; Sigel and Sigel, 2000). However, consumption of high Mn concentrations may cause severe adverse health effects such as a neurodegenerative disorder (Levy and Nassetta, 2003; Sandilyan and Kathiresan, 2014; Singh et al., 2010), cardiovascular toxicity (Jiang and Zheng, 2005), and liver damage (O’Neal and Zheng, 2015). In marine coastal ecosystems such as estuaries, few studies reported Mn as a potentially toxic element since the concentrations are generally low in these ecosystems (Hadlich et al., 2018; McKinley et al., 2019). However, recent studies worldwide, motivated by increasingly large inputs of Mn from human activities, such as mining activity, mining waste, and urban waste, have reported Mn as a potential contaminant for several aquatic species and, thus, its toxicity (Gabriel et al., 2020a; Harford et al., 2015; McKinley et al., 2019; Squadrone et al., 2016; Summer et al., 2019).

The risks associated with Mn are dynamic within estuaries since its bioavailability is driven by oscillating redox and acid-base conditions, leading to many possible fates (e.g., precipitation, adsorption, solubilization) and interactions with other mineral phases (carbonates, oxides, sulfates, and sulfides) (Du Laing et al., 2009b; Namgung et al., 2020; Otero et al., 2009; Thamdrup et al., 1994). Previous studies reported the precipitation of Mn with carbonates under anoxic conditions (Rhodochrosite; Lee et al., 2011; Zachara et al., 1991); at the same time, manganese sulfides (MnS), despite the restricted range of geochemical conditions favorable for their formation and stability, have also been reported under anoxic conditions (Lee et al., 2011; Stumm and Morgan, 1996). The oscillating redox conditions common in estuarine soils may also lead to Mn interactions with Fe oxyhydroxides (Mn associated with Fe; Burdige, 1993; Thamdrup et al., 1994) and formation of Mn oxides (e.g., birnessite; (Postma, 1985; Jacobsite; Burdige, 1993) during oxidizing periods. Thus, the Mn biogeochemical cycle is widely reported as closely associated with the Fe biogeochemical cycle (Burdige, 1993; Lewis and Landing, 1991; Slobodian and Badoz, 2019; Van Cappellen et al., 1998). In fact, these elements are involved in a wide spectrum of biogeochemical pathways such as mineral dissolution, microbial processes, flux-control of trace metals, the formation of a wide array of highly reactive solid phases (Fe and Mn oxy-hydroxides), and the biogeochemical cycles of other major elements (e.g. carbon, sulfur, and phosphorus; Borch et al., 2010; Duckworth et al., 2009). Therefore, coupled studies of Fe-Mn are crucial to advancing our understanding of a wide range of elemental cycles coupled with mechanisms that contribute to environmental contamination, particularly by manganese.

In 2015, a large-scale mine tailings dam disaster occurred in Brazil releasing 43 million m^3^ of Fe-rich tailings into the Rio Doce, one of the country’s largest river basins. The tailings were transported approximately 600 km downstream and reached the estuary and the ocean 16 d after the dam collapse (de Gomes et al., 2017; Queiroz et al., 2021). The disaster represents one of the largest failures of a tailings dam ever recorded and the largest environmental disaster in Brazil’s mining history (Carmo et al., 2017), also killing 19 people and causing extensive ecological (e.g., soil and water pollution; Bernardino et al., 2019; Gabriel et al., 2020b; Queiroz et al., 2018), economic, social and cultural damages (Fernandes et al., 2016).

In addition to the high content of Fe, previous studies have reported the presence of trace metals (e.g, Cu, Cr, Ni, and Zn) in the estuarine soils following the mine tailing contamination (Gomes et al., 2017; Queiroz et al., 2018). These metals arrived in the Rio Doce estuary associated with mine tailings, which are predominantly composed of Fe oxyhydroxides that have strong affinity with metals (Gabriel et al., 2020a; Queiroz et al., 2018). Among the reported elements, Mn does not have a threshold for soil quality according to Brazilian legislation for contaminated soil (CONAMA, 2009), but may pose different risks to the estuarine environment due to its naturally high affinity to Fe and its fast dynamics in redox active environments (Andreji and Stráňai, 2007; Gabriel et al., 2020b; Kennish, 2002; McKinley et al., 2019).

It is not surprising that Mn has not yet been reported as a contaminant in the Rio Doce estuary since Mn contamination in estuarine ecosystems is often overlooked (McKinley et al., 2019; Pinsino et al., 2012). We hypothesize that due to the redox environment in the Rio Doce estuarine soils, the Fe oxyhydroxides will act as sources of Mn leading to a potential risk of Mn contamination. Accordingly, the objective of this study was to evaluate the potential risk of Mn contamination in the Rio Doce estuary two years after the tailings arrival. We assessed the geochemical mechanisms controlling Mn bioavailability coupled to Fe dynamics in a redox active environment with Fe enrichment, to serve as a basis for public policies in coastal wetlands with potential risk of Mn contamination. Thus, the Rio Doce estuary offers a unique framework to evaluate the role of Fe oxyhydroxides controlling the Mn cycle and the environmental health in estuarine ecosystems.

## 2. Materials and methods

### 2.1. Site description

The Rio Doce estuary (19°37′51.45′′S, 39°48′54.62′′W) is located in SE-Brazil with a humid tropical climate classified as *Am*, according to the Köppen-Geiger climate classification system, presenting two distinct seasons including dry winters (from April to September) and wet summers (from October to March; Alvares et al., 2013; Bernardino et al., 2015). *Eleocharis acutangular*, *Typha domingensis*, and *Hibiscus tiliaceus* are the dominant local plant species. The Rio Doce basin is within the Brazilian Iron Quadrangle, a region rich in rocks such as itabirites with highly concentrated ores of Fe, Mn, and Al and where mining activity (e. g., iron, gold, bauxite, and manganese) is of great economic importance (ANA, 2020; de Rodrigues et al., 2014; Silva et al., 2017). In 2015, the Rio Doce estuary was the final destination of the Fe-rich mine tailings that were dumped into the river basin after the Mariana mining dam collapse (Gomes et al., 2017).

### 2.2. Sample collection

**Fig. 1.**
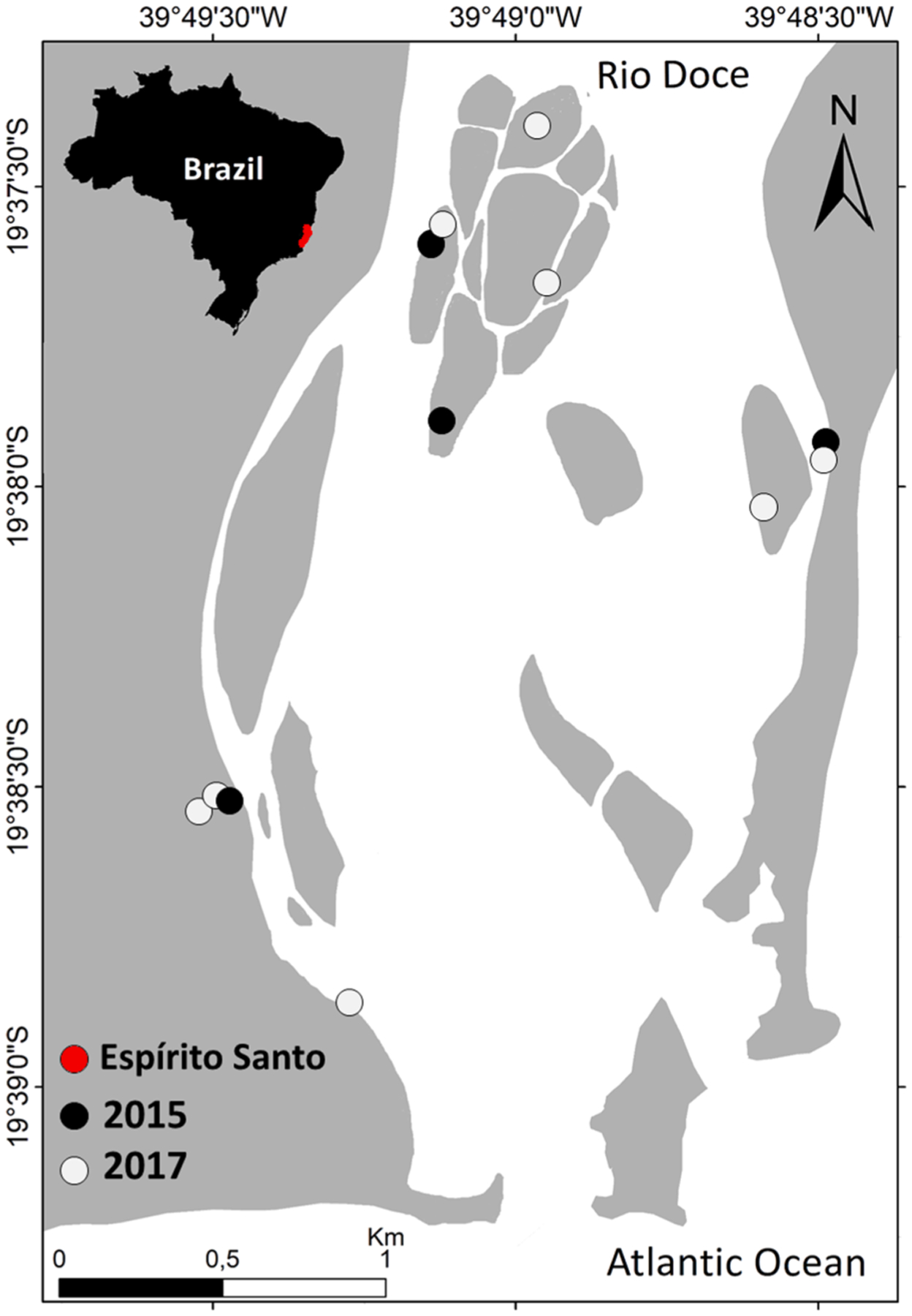
Location of soil sampling sites in 2015 and 2017 in the Rio Doce Estuary, Reĝencia, Espírito Santo, Brazil.

The estuarine soils were sampled in 2 periods: (i) in 2015, seven days after the arrival of the tailings to the estuary (for more details see Queiroz et al., 2018); and (ii) two years after the dam collapse, in 2017, to evaluate possible temporal variations. Samplings were performed during the same season in both campaigns (i.e., the wet season). Soil cores were collected using PVC tubes attached to a flooded soil sampler at four different sites in 2015. In 2017, cores were collected from eight sites, including the four sites sampled in 2015, to achieve a more comprehensive representation of sites affected by tailings deposition (Fig. 1). Additionally, a mine tailings sample collected inside the dam at the site of rupture located at Bento Rodrigues City, Minas Gerais, was donated by the Brazilian National Mining Agency (Aĝencia Nacional de Mineração – ANM) and analyzed to determine the total Mn contents.

After sampling, cores were hermetically sealed and transported upright to the laboratory. In the laboratory, cores were sectioned at different depths depending on the year they were collected. In 2015 the samples were sectioned into 0–3, 3–5, 5–10, and 15–30 cm sections, totaling 21 samples (n = 21); whereas the samples collected in 2017 were sectioned into 0–3, 3–5, 5–10, 10–15, 15–20, 20–25, 25–30, and 30–35 cm intervals (total of 60 samples), to obtain a higher resolution of changes along the soil profiles.

Redox potential (Eh) and pH values of soils were determined in the field using portable meters and an electrode system using samples collected with a semi-open cylindrical soil auger. The pH meter was calibrated at pH 4.0 and 7.0 with standard solutions and the Eh meter measurements used a calomel reference electrode (+244 mV S.H.E.).

Water samples were collected in both years (n = 10 and n = 20 for the years 2015 and 2017, respectively), filtered (pore size 0.45 μm), and acidified with 0.45 mol L^−1^ HCl (trace metal grade) for the determination of dissolved Mn concentration. Water samples were collected from boreholes made with PVC tubes during soil core collection representing the pore water from the saturated soil zone that naturally drains towards the river. The total Mn content in all water samples was determined using ICP-OES (Thermo Scientific – iCAP 6200).

### 2.3. Total contents and sequential extraction of Fe and Mn

The total contents of Fe were obtained from estuarine soil samples and total Mn contents were obtained from both estuarine soil and mine tailings from inside the dam. The total contents were determined using ICP-OES (Thermo Scientific iCAP 6200) after tri-acid digestion in a microwave (HF + HCl + HNO_3_; USEPA, 1996).

Sequential extraction of Fe and Mn was performed on soil samples using a combination of methods proposed by Tessier et al. (1979), Huerta-Diaz and Morse (1990), and Fortin et al. (1993) to determine 6 operationally distinct fractions:

1. Exchangeable and soluble Fe and Mn (EX): extracted with 30 mL of 1 mol L^−1^ MgCl_2_ solution at pH 7.0 at 4 °C, agitated continuously for 30 min.
2. Fe and Mn bound to carbonates (CA): obtained with 30 mL of 1 mol L^−1^ NaOAc at pH 5.0, agitated for 5 h.
3. Fe and Mn bound to ferrihydrite (FR): extracted with 30 mL of 0.04 mol L^−1^ hydroxylamine + acetic acid (25% v/v) solution by shaking for 6 h at 30 °C.
4. Fe and Mn bound to lepidocrocite (LP): extracted with 30 mL of 0.04 mol L^−1^ hydroxylamine + acetic acid (25% v/v) solution by shaking for 6 h at 96 °C.
5. Fe and Mn bound to crystalline Fe oxyhydroxides (mainly goethite; CR): extracted with 20 mL of 0.25 mol L^−1^ sodium citrate + 0.11 mol L^−1^ sodium bicarbonate with 3 g sodium dithionite, agitated for 30 min at 75 °C.
6. Fe and Mn associated with pyrite (PY): extracted with concentrated HNO_3_ (2 h agitation) previously treated with 10 mol L^−1^ HF for silicates removal.

### 2.4. X-ray absorption spectroscopy

X-ray absorption spectra from the five most representative estuarine soil samples were obtained using beamline 7–3 at the Stanford Synchrotron Radiation Lightsource. Spectra were collected at the Mn K-edge using a Si (220) crystal set with orientation φ = 90°, with the beam detuned by 50% at 7500 eV. Soil samples had been previously dried in a 95% N_2_: 5% H_2_ atmosphere before being ground using a mortar and pestle. Samples were packed into aluminum sample holders and sealed with 0.5 mil Kapton tape prior to XAS analysis under ambient conditions. For each sample, two replicate scans were obtained, and beam damage was avoided by moving fresh sample into the beam path. An inline Mn foil was used as a reference for all scans.

Calibration, normalization, and merging of replicate scans was performed using the Demeter package (version 9.26) (Ravel and Newville, 2005) with Larch running as a backend (Newville, 2013) on Windows 10. The average Mn oxidation number (AMON) of the Mn in each sample was obtained through linear combination fitting analysis of the Mn X-ray absorption near-edge structure (XANES) spectra and was performed in Athena (Ravel and Newville, 2005) using the Combo method of Manceau et al. (2012). In all cases, reference spectra from 12 pure-valent Mn species were used to perform unconstrained linear fits. Any reference yielding a negative loading was progressively removed on a per-sample basis and re-added to the reference list before fitting the next sample.

The fraction of Mn^II^, Mn^III^, and Mn^IV^ and AMON were calculated from the fits according to Manceau et al. (2012). A paired-sample *t*-test was used to assess the difference between the means of the AMON data corresponding to soil samples. For the assessment of the relative fraction of Mn distributed between adsorbed Mn^II^ and Mn oxide phases, linear combination fitting of EXAFS was used. For this technique, three Mn^II^ standards, 6 Mn^III,IV^ oxides, and 2 Mn oxides containing Mn^II^ were used to fit the spectra. EXAFS references were a mixture of spectra collected in-house and those obtained by Santelli et al. (2011). The fractional weights for all oxide phases were combined and compared to the combined weight of the Mn^II^ standards prior to comparison of means through a paired-sample *t*-test. Fits were conducted between K = 3 and K = 11. XANES and EXAFS data from the standards used in all linear combination fitting, as well as the XANES linear combination fittings and data are available in the SI.

### 2.5. Diffuse reflectance spectroscopy

Diffuse reflectance spectroscopy (DRS) was used for the mineralogical characterization of soil samples. DRS spectra were measured from 300 to 800 nm at 1 nm increments with a 110 mm integrating sphere using a Varian Cary 5 Spectrophotometer. The results were transformed using the Kubelka Munk function to calculate the second derivative. Spectra from surface soil samples (0–3 cm, from 2015 and 2017) and subsurface samples (30–40 cm, depth with lower tailings deposition influence) from 2017 were obtained to compare the estuarine soil composition after tailings deposition as well as mineralogical changes over time.

### 2.6. Fish collection and analyses of metal contents in tissues

To assess the risk of Mn contamination to local wildlife, two fish species were collected in 2017 using a bottom Otter Trawl, at random locations in proximity to the soil sampling sites (Fig. 1). *Cathorops spixii* (Agassiz, 1829) (Madamango sea catfish; n = 15) and *Genidens genidens* (Valenciennes, 1839) (Guri sea catfish; n = 18) are estuarine species which spend their entire life cycle associated with bottom sediment. In addition, these species have been used previously as bioindicators of pollution and are an important food resources for the local population (Azevedo et al., 2009; Pinheiro and Joyeux, 2007).

After collection, fish were immediately frozen until dissection in the laboratory. Fish liver and axial muscle tissues were dissected and stored at −80 °C until quantification of total metal contents. The total contents of Mn and Fe were determined using approximately 100 mg of dried sample (muscle or liver) weighed in sterile polypropylene tubes, followed by the addition of 1.0 mL of double-distilled HNO_3_. The blanks containing only 1.0 mL of double-distilled HNO_3_ were prepared in triplicate. The DORM-4 (Dogfish muscle – National Research Council, Canada) Certified Reference Material (CRM) was used for quality control. The samples, blanks, and CRM were left in contact with HNO_3_ for approximately 12 h overnight then heated for digestions the following morning on a digester block for 4 h at approximately 100 °C. The closed tubes were monitored hourly with manual pressure relief when necessary. After heating, the samples, CRM, and blanks were left to cool until room temperature and made up to appropriate volumes with ultra-pure water (resistivity > 18.2 MΩ). The Mn and Fe quantification were performed by ICP-MS using an ICP-MS ELAN DRC II (Perkin-Elmer Sciex, Norwalk, CT, USA). ^103^Rh was used as the internal standard at 20 μg L^−1^.

### 2.7. Contamination factor determination

The contamination factor (Cf) was used to evaluate the Mn contamination at Rio Doce estuary, using as a background value the total Mn contents in the soils 11 d before the tailings arrival reported by Gomes et al., (2017). The Cf is a ratio between the content of an element in a soil sample and the background content of the same element at the studied site (Hakanson, 1980), following the equation Cf = Cs / Cb, where Cs is the soil content of Mn in 2017 and Cb is the Mn background value. According to Hakanson (1980), the following interpretations are suggested for the Cf value: Cf < 1, low; 1 to < 3, moderate; 3 to < 6, considerable; and > 6, high contamination.

### 2.8. Statistical analyses

The Fe and Mn total contents in soil and water samples were assessed with a non-parametric Kruskal–Wallis (*p* < 0.05) test to assess differences between 2015 and 2017, whereas the Fe and Mn contents in fish muscles and livers were analyzed with a non-parametric Friedman test at the 5% significance level with multiple pairwise comparisons (Reimann et al., 2008; XLSTAT version 2014.5.03). Non-parametric statistical tests are appropriate for non-normal distributions and rely on fewer assumptions, which make them more robust for environmental data (Reimann et al., 2008). The correlations between the total of Fe and Mn in soil were determined by calculating Spearman’s correlation coefficients (*r*) as this method does not assume a normal distribution.

## 3. Results

### 3.1. Physicochemical conditions, Fe and Mn total contents and fractionating

**Fig. 2.**
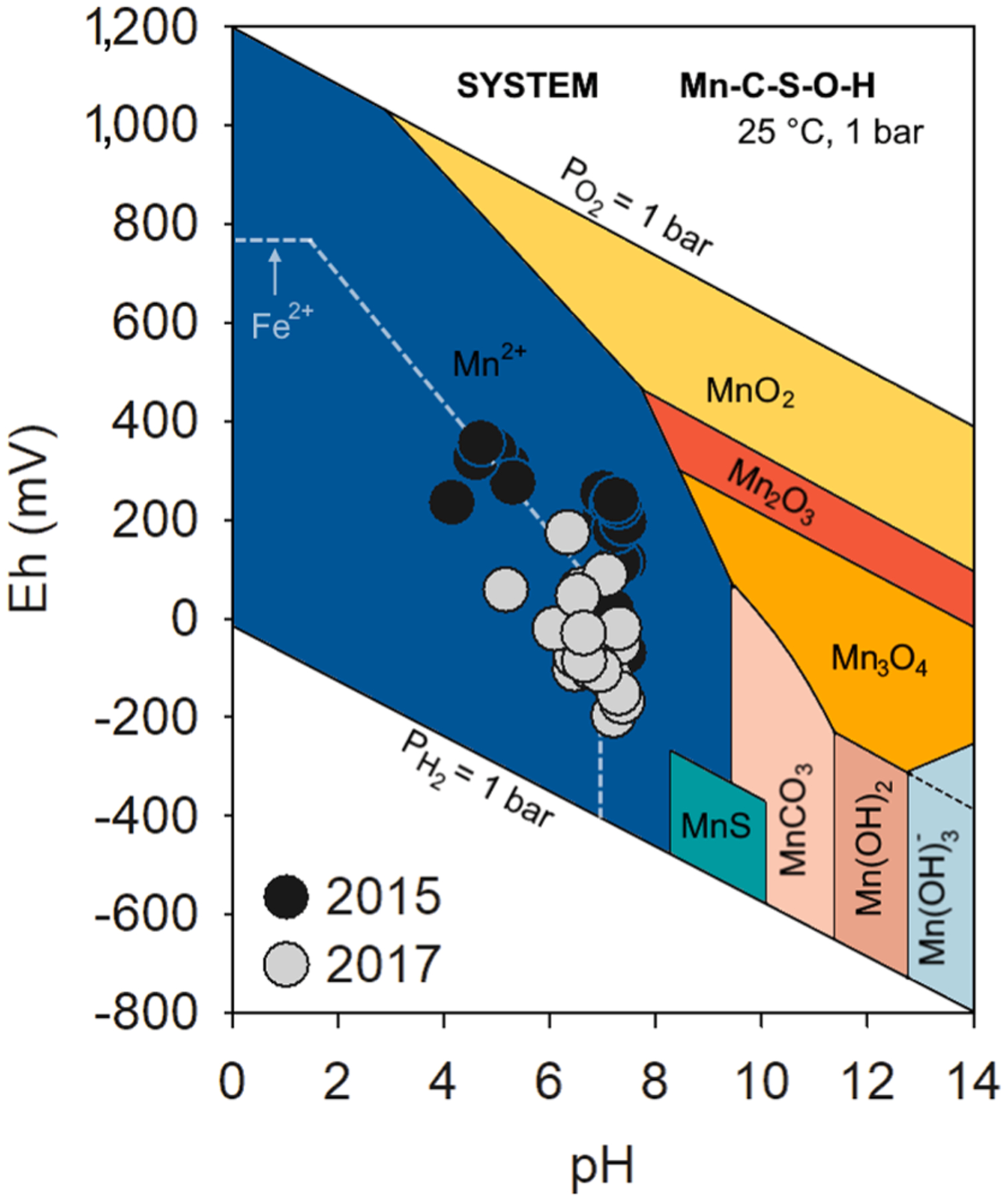
Eh-pH diagram (system Mn-C-S-O-H) with the data for the studied soils in both years. The gray dashed line indicates the Fe^2+^ stability field on the system Fe-C-O-H. The Eh-pH diagram was adapted from Brookins (1988).

The Eh and pH values in 2015 were on average + 218 ± 116 mV and 6.2 ± 1.3, respectively. In 2017, the pH remained close to neutral (average 6.7 ± 0.5) but the Eh values decreased considerably with a mean of −47 ± 83 mV (Fig. 2).

**Fig. 3.**
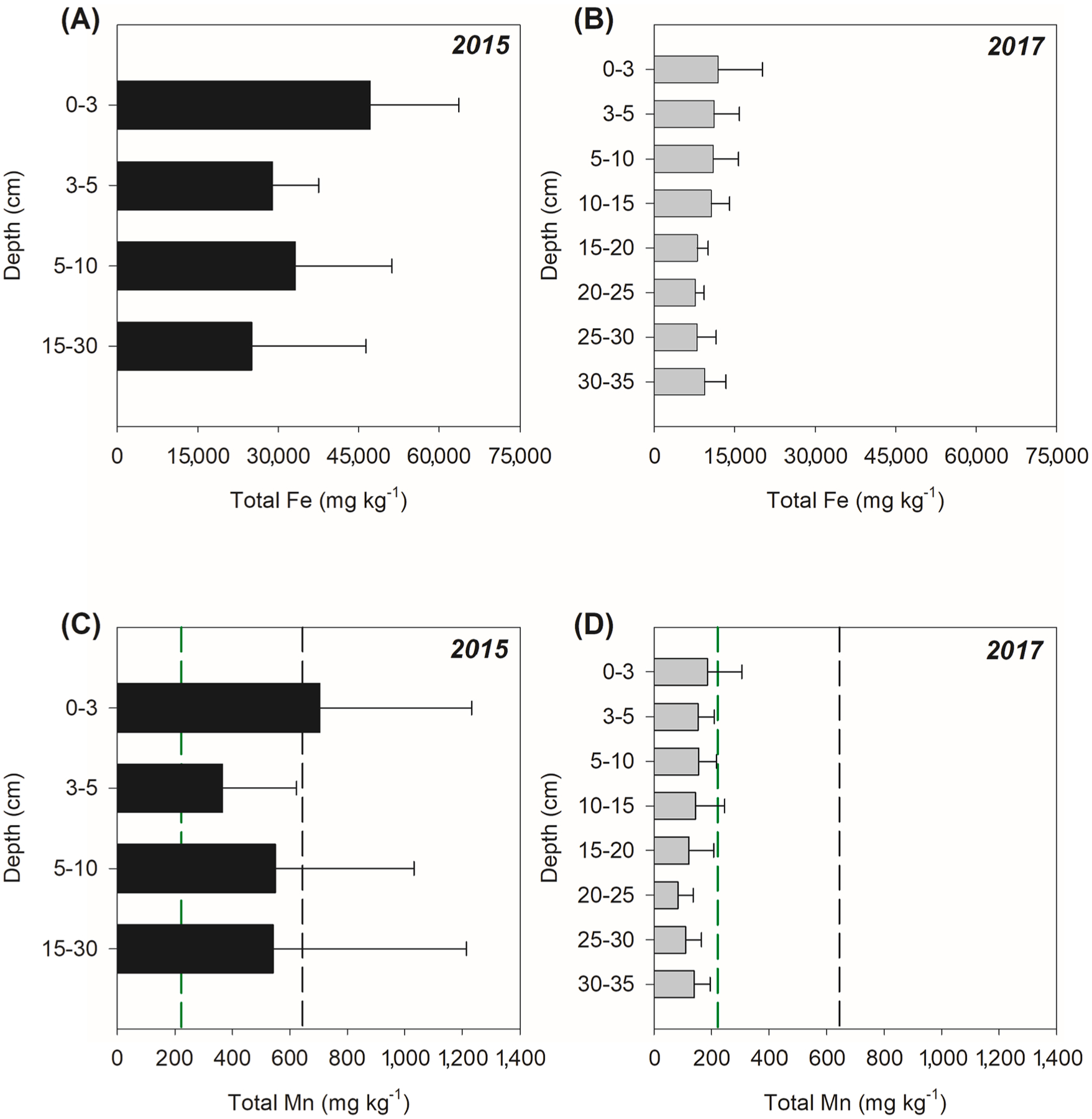
Total Fe and Mn contents of Rio Doce estuarine soils collected in 2015 and 2017. The black dashed line indicates the contents of the total Mn in tailings from inside the Fundão dam. The green dashed line indicates the Mn contents in Rio Doce estuarine soil, prior to mine tailings arrival according to Gomes et al., (2017).

The total Mn content in mine tailings from inside the Fundão Dam was on average 644 ± 241 mg kg^−1^ whereas in the estuarine soil, in 2015, higher total Fe (47,133 ± 16,538 mg kg^−1^) and Mn (704 ± 529 mg kg^−1^) contents were measured in the surface soil layers (0–3 cm), the soil layer most affected by tailings deposition (Fig. 3). Two years later, the mean total Fe and Mn concentrations decreased by 75% and 74% respectively across all soil depths. In 2017, the highest Fe and Mn contents were still found in the upper 0–3 cm (11,997 ± 8,239 mg kg^−1^ and 186 ± 120 mg kg^−1^ respectively), followed by a decrease of both elements with soil depth (depths > 3 cm; Fig. 3). The contamination factor (Cf) using the Mn content in the 0–3 soil layer just after the tailing arrival (in 2015) was 3.2 indicating considerable contamination levels, whereas in 2017 the Cf decreased to 0.84 indicating low contamination.

**Fig. 4.**
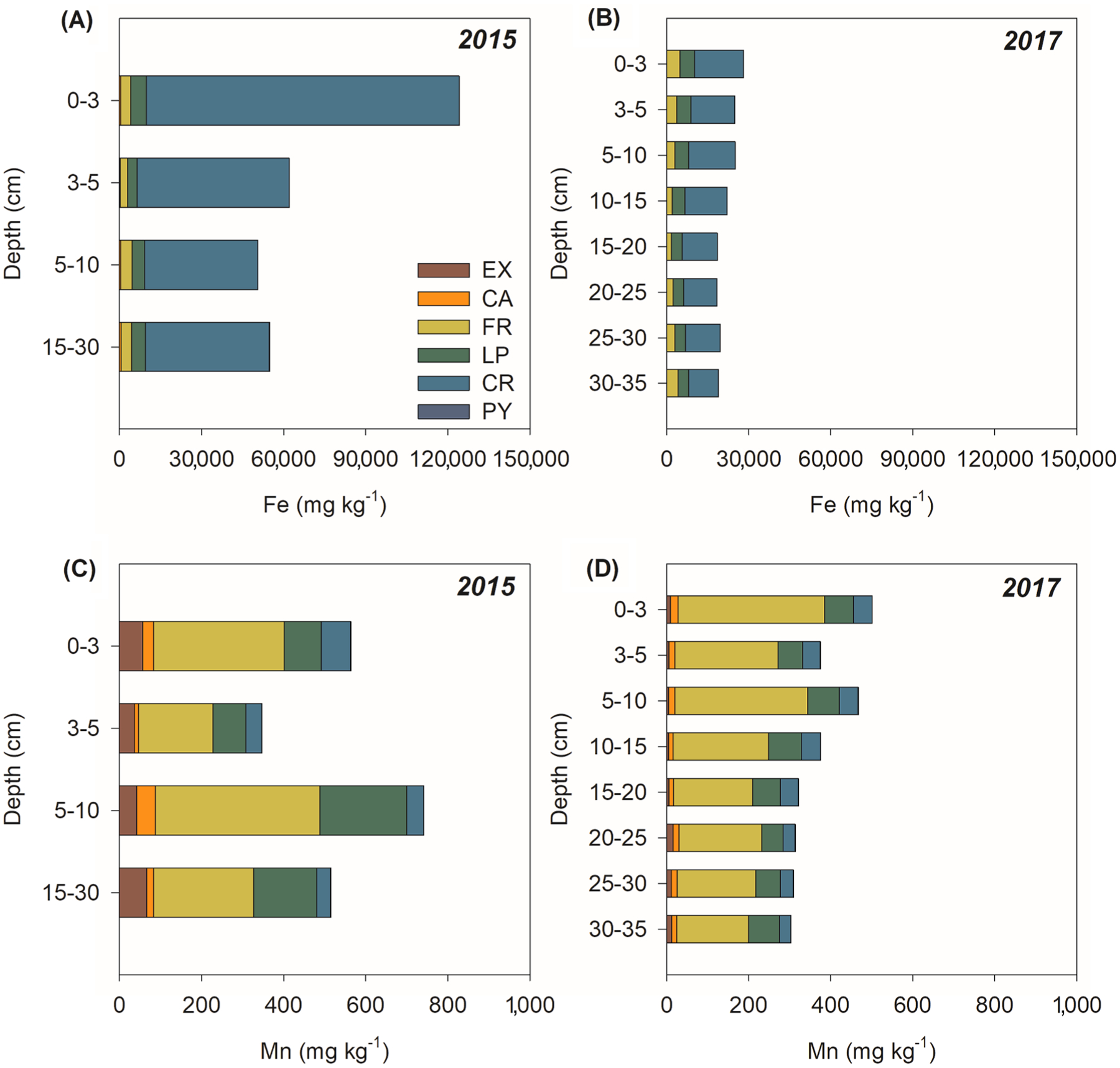
Fe and Mn solid-phase fractionation from Rio Doce estuarine soils in 2015 and 2017. EX: Soluble and exchangeable Fe and Mn; CA: Fe and Mn associated with carbonates; FR: Fe and Mn associated with ferrihydrite; LP: Fe and Mn associated with lepidocrocite; CR: Fe and Mn associated with crystalline oxides; PY: Fe and Mn associated with pyrite.

Solid-phase Fe and Mn fractionation of soils collected in 2015 (Fig. 4) shows Fe mostly held in crystalline Fe oxyhydroxides representing 88% of total Fe (on average: 64,154 ± 45,104 mg kg^−1^), whereas poorly crystalline Fe oxyhydroxides represented only 11% (i.e., average: LP: 4,632 ± 3,635 mg kg^−1^ equivalent to 6%; and FR: 3,645 ± 3,573 mg kg^−1^ equivalent to 5%; Fig. 4). The sum of EX, CA, and PY fractions were approximately 1% of the total Fe.

In contrast, Mn was mainly associated (78%) with poorly crystalline Fe oxyhydroxides (FR: 286 ± 352 mg kg^−1^; LP: 134 ± 171 mg kg^−1^) and to a lesser degree (9%) with the crystalline (CR) fraction (46 ± 27 mg kg^−1^). Soluble and exchangeable (EX) fractions represented 9% (50 ± 78 mg kg^−1^) and Mn associated with carbonates (CA: 25 ± 37 mg kg^−1^) and pyrite (PY: 25 ± 37 mg kg^−1^) represented about 5%.

In 2017, Fe showed a contrasting distribution to that of 2015, with a marked decrease in CR (14,326 ± 2,507 mg kg^−1^; equivalent to 65%) followed by a significant increase in LP (4,493 ± 732 mg kg^−1^; equivalent to 20%), and FR (3,042 ± 1,051 mg kg^−1^; equivalent to 20%). On average, the crystalline Fe oxyhydroxide contents decreased 49,828 mg kg^−1^ when compared to 2015 (Fig. 4). The other fractions (i.e., EX, CA, and PY) remained close to 1% of the sum of all Fe fractions. Similar to 2015, in 2017 Mn was mostly associated with poorly crystalline Fe oxyhydroxides (FR: 241 ± 67 mg kg^−1^; equivalent to 65%; LP: 68 ± 10 mg kg^−1^; equivalent to 18%) and to a lesser extent (11%) associated with crystalline Fe oxyhydroxide phases (CR: 39 ± 8 mg kg^−1^; Fig. 4). In contrast, the soluble and exchangeable Mn fraction decreased considerably (EX: 9 ± 4 mg kg^−1^; equivalent to 2% of total Mn) and Mn associated with carbonates and pyrite represented the less important Mn fractions found in the solid-phase in that year (14 ± 3 mg kg^−1^ and 0.4 ± 0.1 mg kg^−1^, respectively; representing about 4% of Mn).

### 3.2. Spectral reflectance characteristics

**Fig. 5.**
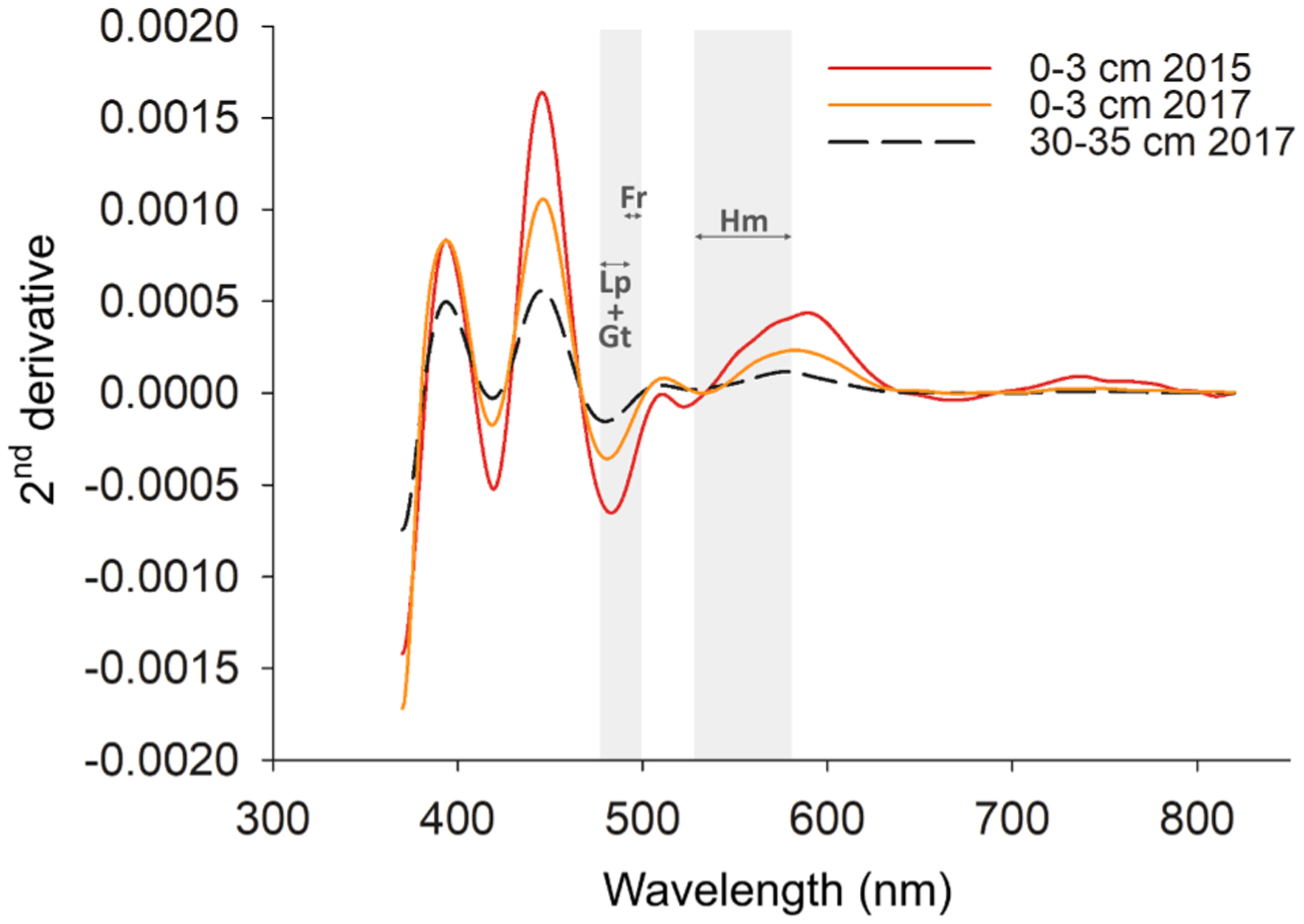
Second-derivative spectra of surface (0–3 cm depth from 2015 and 2017) and sub-superficial soil samples (30–40 cm depth from 2017). Range of crystal field band position for lepidocrocite (Lp) and goethite (Gt) (488 nm), ferrihydrite (Fr; 484–499 nm), and hematite (Hm; 533–588 nm).

The DRS spectra corroborated the solid-phase fractionation and indicated a greater presence of both high- and low- crystallinity Fe oxyhydroxides in the surface soil layers (i.e., 0–3 cm) in both years. Deeper soil layers (30–35 cm) were less influenced by mine tailings with much smaller quantities of Fe oxyhydroxides (Fig. 5) as seen by the lower intensity of bands of iron oxides (Ji et al., 2002). It is noteworthy that band intensities changed over time, with a lower intensity of bands in 2017 corroborating a loss in Fe oxides (Figs. 3 and 4). In fact, the shift in the intensity of bands between 485 and 490 nm indicates a decrease of both lepidocrocite and goethite (absorption band at 488 nm) with time. The same patterns are observed for the absorption bands of ferrihydrite (seen between 484 and 499 nm; Scheinost, 1998) and hematite (absorption band shown at 533–588 nm; Hu et al., 2016). The Fe fractionation analyses (Fig. 4) can aid in distinguishing the relative contributions of the different iron forms to the DSR bands (Scheinost et al., 2001; Schwertmann and Taylor, 1989).

### 3.3. XANES and EXAFS characterization

While Mn fractionation and acid digestions can quantify the total and relative mass of Mn in samples, X-ray absorption analyses including XANES and EXAFS provide Mn oxidation state and coordination information. Mn K-edge XANES showed that more than half of the Mn in the soil solid phase was reduced (52%; Table 1) in the soil surface samples (0–3 cm) from 2015 which represents the readily exchangeable or adsorbed Mn fractions (Tebo et al., 2004), while the abundance of Mn^III^ and Mn^IV^ were 32% and 16%, respectively (Table 1).

**Fig. 6.**
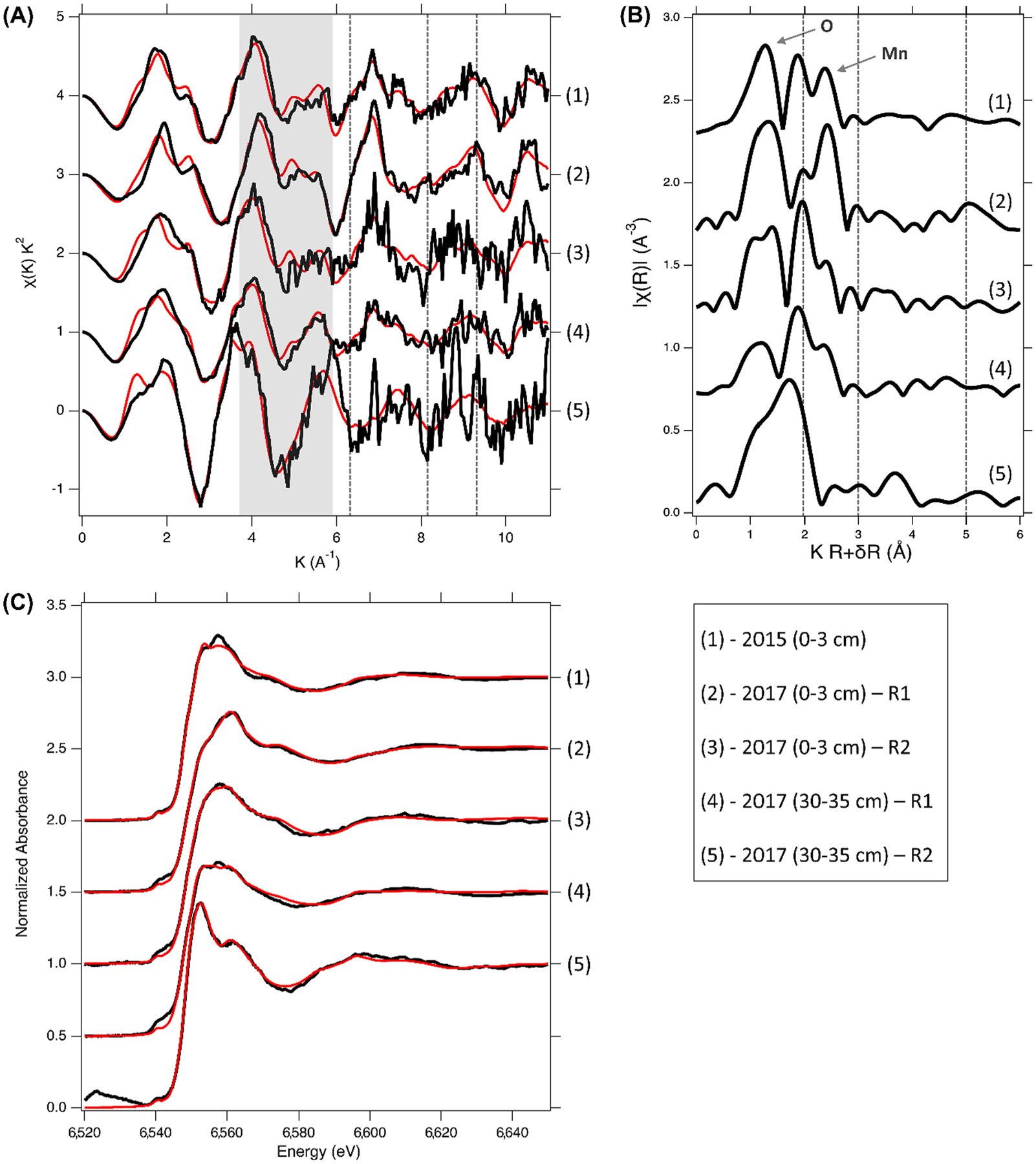
Mn K-edge EXAFS of soil samples from 2015 and 2017 collected at two depths (0–3 cm and 30–35 cm). (A) Shaded area highlights the characteristic “staircase” feature of the EXAFS indicative of phyllomanganate presence; the vertical dashed lines mark the shoulder feature at 6.5 Å^−1^ and the antinodes at 8.2 and 9 Å^−1^. (B) Pseudo-radial structure functions of the Mn EXAFS; vertical lines mark features at 2 Å, 3 Å and 5 Å; the arrows point to the nearest Mn-O and Mn-Mn shells. (C) A comparison of the Mn XANES from 2017 soil samples with the soil surface sample from 2015. Data is shown in black, model generated from linear combination fitting is shown in red.

In 2017, Mn EXAFS analysis shows that surface soil samples (0–3 cm) had a higher concentration of Mn^III^ and Mn^IV^ than soils from 2015, where these oxidized forms of Mn were present as phyllo- and tectomanganates (Fig. 6). In contrast, the corresponding subsurface (i.e. 30–35 cm) soils showed greater concentrations of the Mn^II^ (Table 1).

Mn K-edge EXAFS of the surface soil samples from 2017 displayed the characteristic phyllomanganate (e.g. birnessite; δ-MnO_2_) “staircase” feature between 4 and 6 Å^−1^ (Fig. 6). Extensive corner-sharing cation octahedra dispersed in the interlayer region (i.e. Mn^II^, Mn^III^, Zn, Ni) give rise to the shoulder development along the rising edge of the antinode at 6.4 Å^−1^ (Fig. 5); the corresponding peak at ~ 3 Å is also indicative of the presence of Mn^III^ or Mn^IV^ (Manceau et al., 2002; Marcus et al., 2004; Toner et al., 2006). In addition, the absence of a defined antinode at 8.1 Å^−1^ in any of the samples suggests the presence of tectomanganates, such as pyrolusite (MnO_2_), as well as Mn^III^-rich octahedral sheets in the phyllosilicate minerals present in the 0–3 cm soil layer soil from 2017 (Marcus et al., 2004; Webb, 2005; Zhu et al., 2010).

### 3.4. Mn in water and in fish tissues

**Fig. 7.**
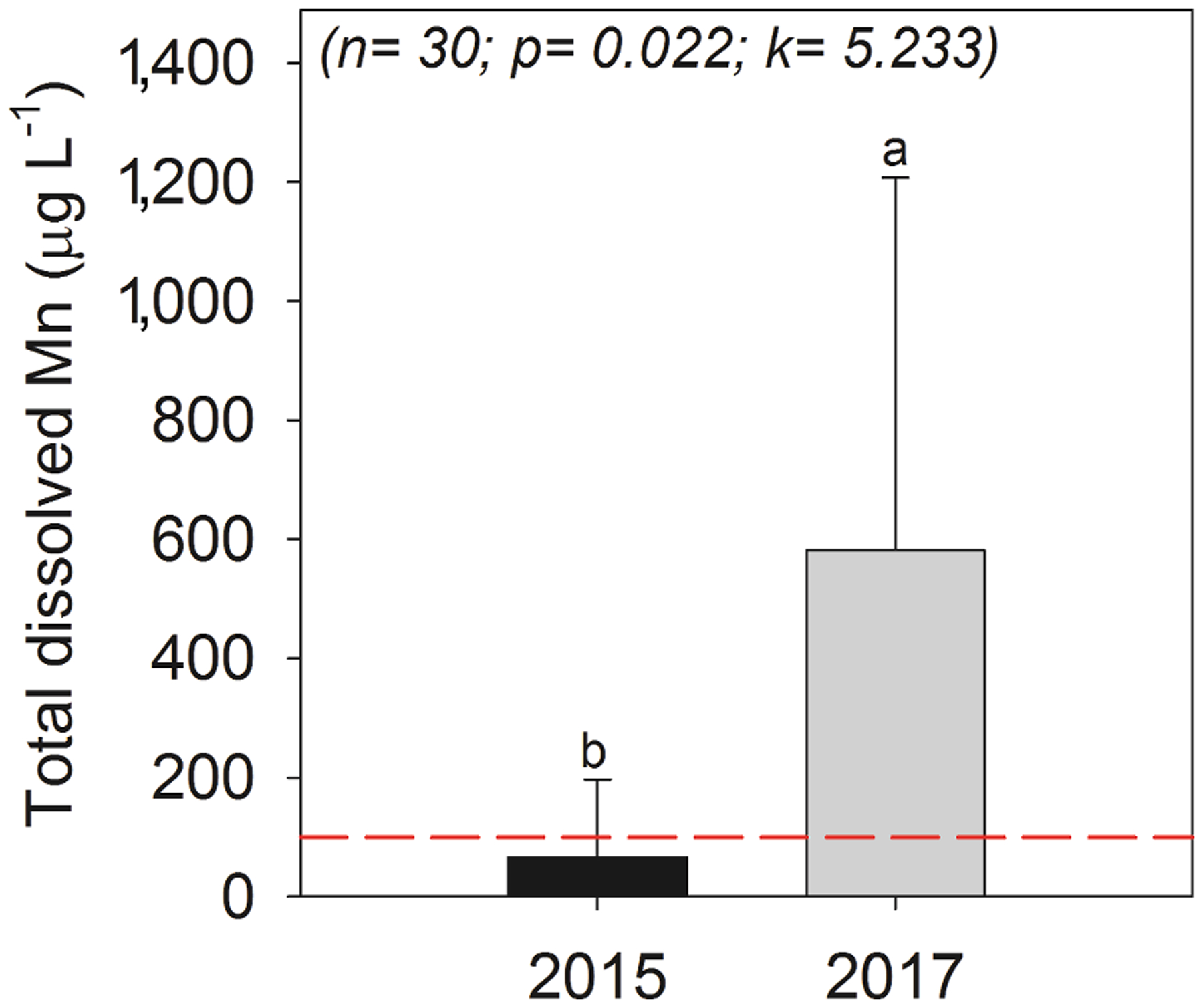
Total concentration of dissolved Mn in the Rio Doce estuary water sampled in 2015 and 2017. The red dashed line indicates the threshold according to the Brazilian water quality guidelines for brackish water (100 μg L^−1^; CONAMA, 2005). The different lowercase letters indicate a significant difference between the variables as determined by the Kruskal-Wallis test at the 5% probability level.

The mean total dissolved Mn concentration in water samples collected in 2015 was 66 ± 130 μg L^−1^, whereas in 2017 the average concentration increased 9-fold to 582 ± 626 μg L^−1^ (Fig. 7). The 2017 concentrations are 5 times higher than the threshold outlined by the Brazilian water quality guidelines for brackish waters (100 μg L^−1^ for inorganic constituents in brackish waters without chronic toxic effects on organisms; CONAMA, 2005). In comparison, the concentration of dissolved Mn in 2017 was higher than the threshold for chronic contamination in marine water according to the National Oceanic and Atmospheric Administration, USA (100 μg L^−1^; NOAA, 2008).

**Fig. 8.**
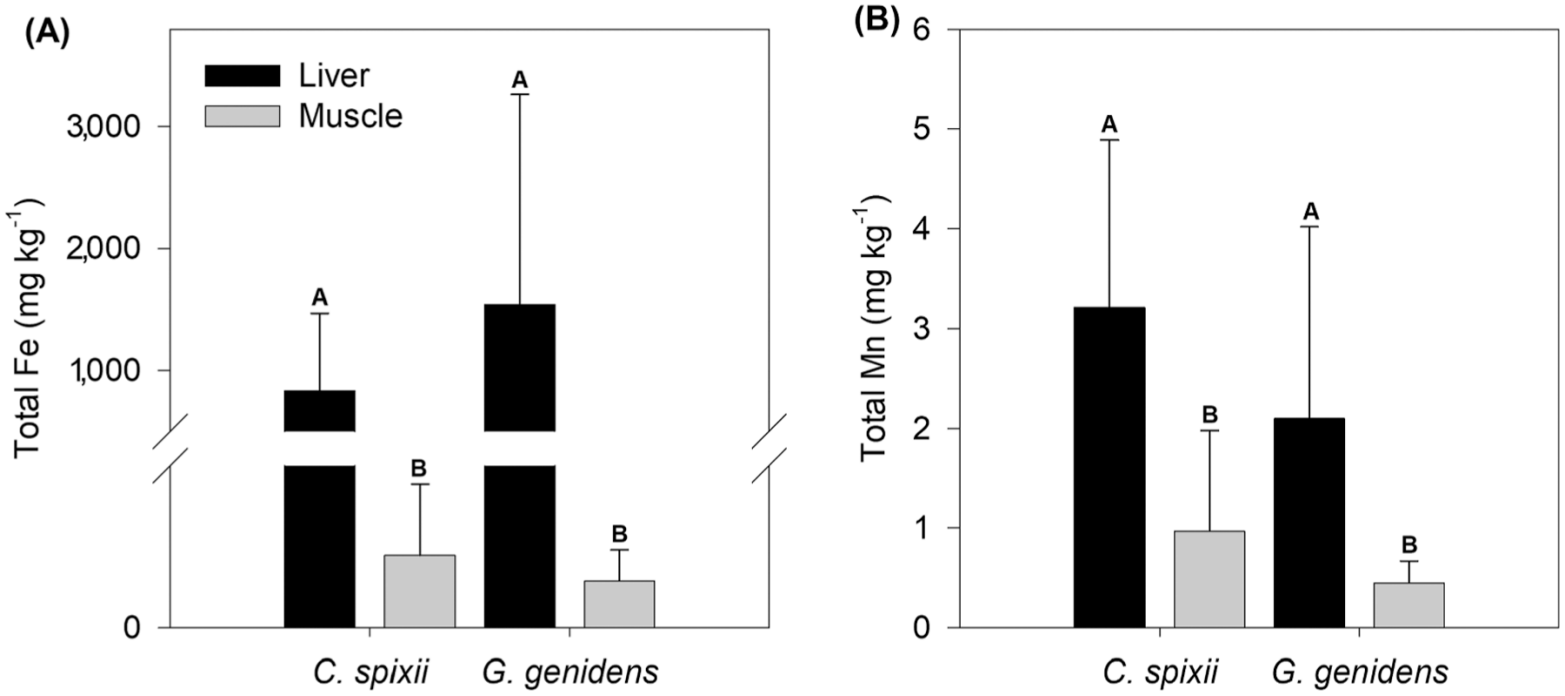
Total contents of Fe (A) and Mn (B) in the liver and muscle of *Cathoropus spixii* and *Genidens genidens* from Rio Doce. Labelled bars with uppercase letters (A and B) indicate groups between which statistical differences among species or tissues exist at the 5% probability level using the non-parametric Friedman test.

The Fe and Mn contents in fish liver and muscle tissues were significantly different, but did not differ between species (Fig. 8). Higher concentrations of Fe and Mn were observed in the liver than in the muscle (Fig. 8). The mean Fe content in the liver was 830.1 ± 638.0 mg kg^−1^ and 1,541.4 ± 1,725 mg kg^−1^ respectively in *Cathoropus spixii* and *Genidens genidens*, whereas the mean Mn contents were 3.2 ± 1.7 mg kg^−1^ and 2.1 ± 1.9 mg kg^−1^. In the muscle, Fe and Mn concentrations in *Cathoropus spixii* were 26.8 ± 26.4 mg kg^−1^ and 1.0 ± 1.0 mg kg^−1^, respectively; whereas in *Genidens genidens* mean Fe and Mn contents in muscles were 17.4 ± 11.4 mg kg^−1^ and 0.5 ± 0.2 mg kg^−1^, respectively (Fig. 8). There is no contamination threshold for either Mn or Fe for both studied fish species.

## 4. Discussion

### 4.1. Mining tailing disaster and its impacts on Mn geochemistry

**Fig. 9.**
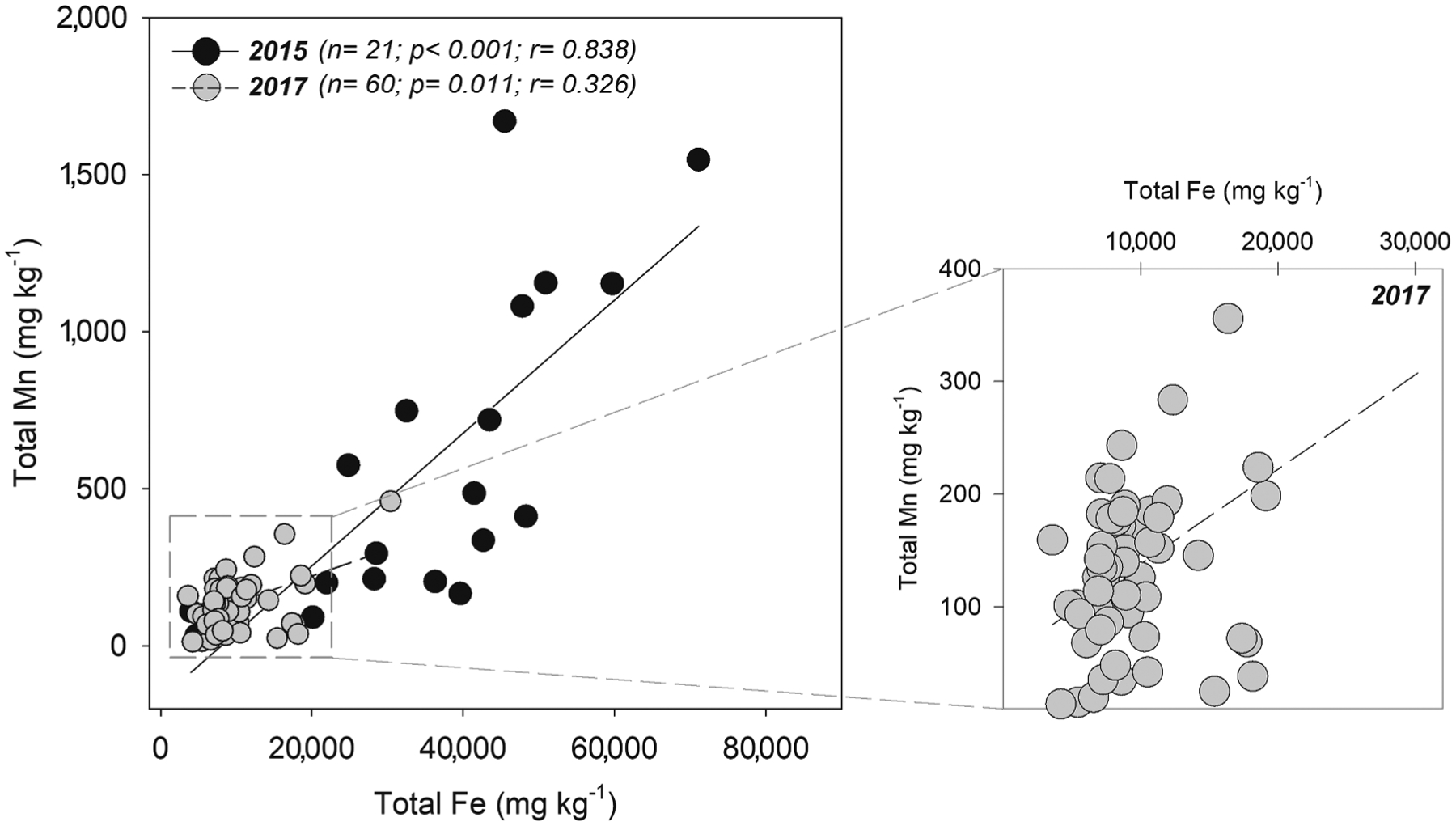
Spearman correlations between total Fe and Total Mn in 2015 and 2017. The right panel shows in detail the spearman correlation between total Fe and total Mn content from 2017 highlighting the loss of correlation with time.

In 2015, because of the Fundão dam rupture, Fe-rich tailings were dumped into the Rio Doce basin and traveled 688 km downriver toward the estuary (Gomes et al., 2017). The tailings were made mostly of crystalline Fe oxyhydroxides which have high metal retention capacity (Cornell and Schwertmann, 2003; Queiroz et al., 2018). Therefore, we hypothesized the tailings may have acted as a Mn sink until its ultimate deposition in the estuary. In addition to the high content of Mn (644 ± 241 mg kg^−1^) in the original tailings from inside the dam (Fig. 3), agricultural activities and pollution from large cities and villages along the basin may have acted as additional Mn sources to the mine tailings transported in the Rio Doce river on its way to the estuary (Queiroz et al., 2021). In the past, studies prior to the Mariana disaster also reported Mn contents ranging from 660 to 2,280 mg kg^−1^ in mine tailings from Samarco’s dams located in the same mining complex as Fundão dam (Pereira et al., 2008). Indeed, the Mn contents in the surface soil layers (0–3 cm) from 2015 (704 ± 529 mg kg^−1^), were on average 3-fold higher than Mn contents 11 d before the disaster (222 ± 13 mg kg^−1^) reported by Gomes et al., (2017) indicating the tailings deposition effects. In this sense, the Cf calculated (3.2) indicates a Mn enrichment in the soil soon after the disaster and considerable contamination. Moreover, the significant positive correlation between Fe and Mn (Fig. 9) in samples collected from 2015 support the arrival of Mn to the estuary in association with the Fe-rich mine-tailings (i.e. adsorbed to Fe oxyhydroxides).

The interaction of Mn with Fe oxyhydroxides in soils has been widely reported due to the energetic favorability of Mn forming mono- and binuclear inner-sphere complexes through reacting with excess structural OH^−^ groups on the Fe oxyhydroxide surface (Ugwu and Igbokwe, 2019; Zhu et al., 2020). In general, Fe oxyhydroxides uptake the Mn^II^ forming virtually irreversible complexes (Coughlin and Stone, 1995; Namgung et al., 2020). Indeed, the EXAFS results showed higher abundance of Mn^II^ in the 0–3 cm depth range from 2015 (Table 1), as well as in the Fe fractionation, which indicates that Mn was mostly associated with Fe oxyhydroxides (FR: 53%; LP: 25%; and CR: 9%). In addition, circumneutral pH and Eh values above + 100 mV recorded in 2015 (Fig. 2) indicate suboxic conditions that are favorable to Fe oxyhydroxide formation (Reddy and DeLaune, 2008).

Once the Fe-rich tailings arrived and were deposited on the soils of the Rio Doce estuary, the tailings were then exposed to redox oscillating conditions caused by tidal flooding and plant activity (Bianchi, 2007; Du Laing et al., 2009a). By 2017, a sharp decrease in Eh (−46 ± 83 mV, on average) was observed compared to measurements in 2015 (+218 ± 116 mV, on average) indicating increasingly anoxic conditions (Fig. 2) (Reddy and DeLaune, 2008; Søndergaard, 2009). The marked decrease in Eh over time is likely due to estuarine plants (i.e., *Eleocharis acutangula*, *Typha domingensis*, and *Hibiscus tiliaceus*) that serve as direct inputs of organic matter (e.g. via dead leaves and roots) while also efficiently trapping particulate organic matter (OM) that is transported downstream. These plants contributing OM inputs coupled with tidal flooding stimulate anaerobic OM degradation and Fe^III^ reduction (Badarudeen et al., 1996; Kristensen et al., 1995; Marín-Muñiz et al., 2014). Additionally, the plant growth enhances the maintenance of settled tailings since plant stems reduce the turbulence kinetics that could lead to physical tailings removal (Jay et al., 2007; Mudd et al., 2010).

**Fig. 10.**
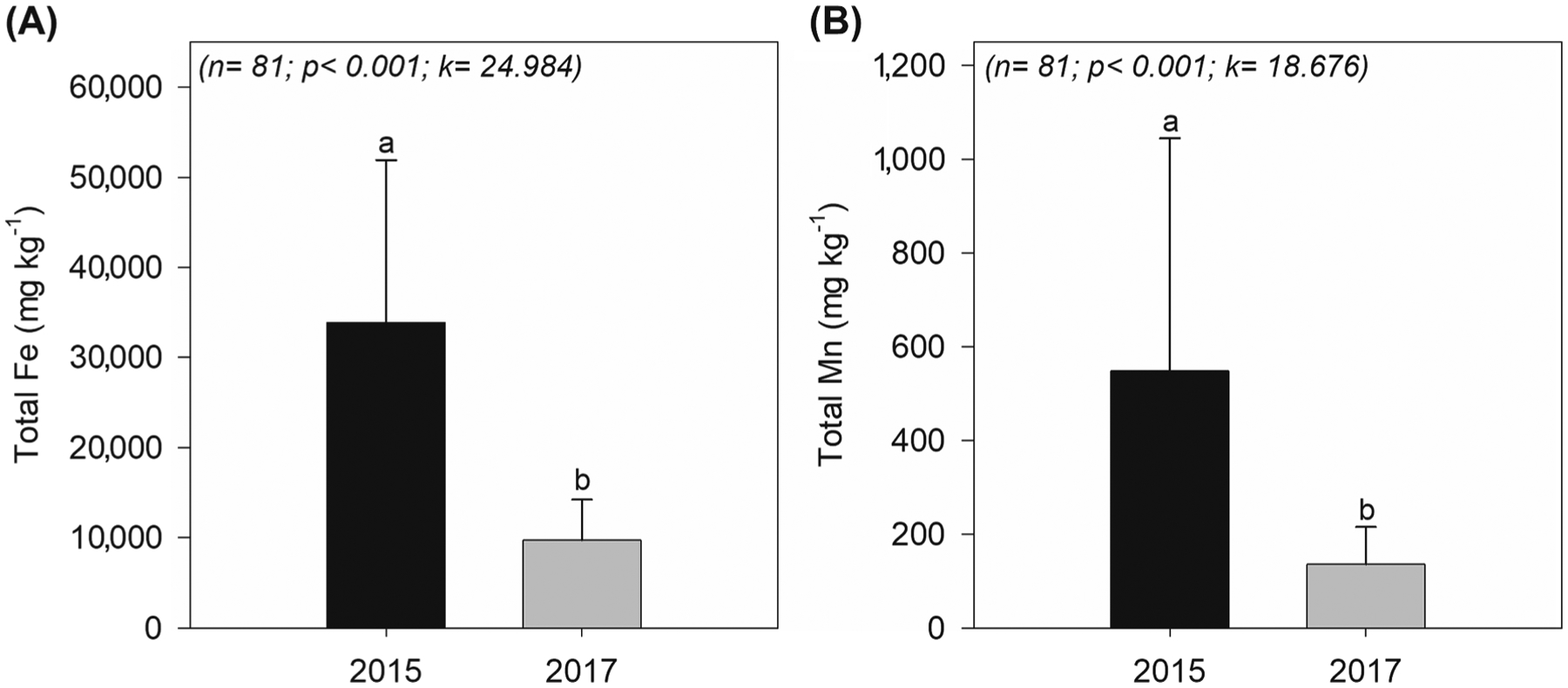
Mean Fe (A) and Mn (B) total soil contents from 2015 and 2017. The different lowercase letters indicate significant differences among the variables using the Kruskal-Wallis test at the 5% probability level.

Thus, Fe oxyhydroxides from the tailings were subjected to a biogeochemical environment highly favorable towards Fe^III^ reduction to Fe^II^ and its subsequent solubilization (Cummings et al., 2000; Xia et al., 2019). In estuaries, the fate of solubilized Fe^II^ following dissimilatory Fe reduction may vary, for instance as precipitation of poorly crystalline Fe oxyhydroxide, uptake by plants, or removal from the estuary into the ocean (Canfield and Kristensen, 2005; Johnston et al., 2011; da Richard et al., 2020). The significant decrease in total Fe in soils collected in 2017 (*r* < 0.001; Fig. 10), mainly at the soil surface, which was the soil layer most affected by tailings deposition, clearly corroborates a massive loss of total Fe through reduction (i.e. reductive dissolution). Additionally, the total Fe loss reflected the significant loss of the CR Fe fraction (Fig. 4) which was also supported by a clear decrease in the goethite and hematite bands of the DRS spectra likely due to their dissolution (Canfield et al., 1993; Lovley et al., 2004).

The total Mn content in the soil from 2017 also showed a significant decrease (*p*-value < 0.001; about 75%) when compared to 2015 data (Fig. 10). Furthermore, the solid-phase fractionation showed that Mn associated with both high and low crystallinity Fe oxyhydroxides decreased on average 25% in 2017 (Fig. 4). Likely, the decrease of soil Mn contents was associated with its release as Mn^II^ in response to Fe oxyhydroxides dissolution. In fact, the association of Mn with high and low crystallinity Fe oxyhydroxides was clearly shown by the Mn K-edge XANES data (Table 1).

Following release and diffusion out of soils, dissolved Mn^II^ had a number of possible fates: 1) be transported further downstream and washed out from the estuary (particularly during the rainy season); 2) be retained on Fe oxyhydroxides; or 3) undergo oxidation followed by precipitation as Mn oxides (Chaudry and Zwolsman, 2008; Otero et al., 2009; Sundby et al., 2003). XANES analysis of soil samples from 2017 (Table 1) shows that a high proportion of Mn had been oxidized (i.e., Mn^III^ and Mn^IV^) in the soil surface layers (i.e. 0–3 cm) corresponding to EXAFS spectra with characteristics attributable to phyllo- and tectomanganates (i.e., Mn oxides; Fig. 5). According to Oldham et al. (2019) Mn^II^ can be quickly oxidized to Mn oxides in estuarine surface soils because O_2_ diffusion in the surface layers is more rapid than at depth.

It is likely during the first two years (2015 to 2017) after the arrival of the tailings that the prevailing conditions in the estuarine soils favored the release of Mn^II^ through reduction of Fe oxyhydroxides, with subsequent transformation of Mn^II^ into poorly crystalline Mn oxides due to redox fluctuations (Fig. 2). Previous studies reported that both dissolved Mn and Fe may re-oxidize or co-precipitate with a variety of different soil minerals (e.g. oxides, carbonates, sulfides) (Du Laing et al., 2009b; Otero et al., 2009). However, by 2017, the redox potential observed in Rio Doce estuarine soils indicated geochemical conditions (i. e., Eh and pH) had become favorable for anaerobic processes including both Fe and Mn dissimilatory reduction (Fig. 2) (Canfield et al., 1993; Lovley et al., 2004). It is widely known that anaerobic microorganisms through microbial reduction of both Fe and Mn may use Mn^III^, Mn^IV^, and Fe^III^ present on minerals as electron acceptors for anaerobic respiration under anoxic conditions (Otero et al., 2009; Patrick and Jugsujinda, 1992). Thus, our results indicate that Mn released during 2017 onwards might have occurred through reduction of both Fe oxyhydroxides and poorly crystalline Mn oxides (Lovley et al., 2004; Postma and Appelo, 2000).

Therefore, an increase in dissolved Mn concentrations is expected given that Mn^II^ is generally more stable against abiotic oxidation by O_2_ than Fe^II^, which can be rapidly oxidized under Eh conditions above + 100 mV and circumneutral pH (Burdige, 1993; Frohne et al., 2011). Although Mn oxides have been formed due to O_2_ diffusion in surface layers, the Mn oxides required very strong oxic conditions (approximately + 900 to + 1000 mV at pH 5) to reach stability against reductive processes (Frohne et al., 2011). In our study, the highest Eh value measured in 2017 was + 174 mV. The Fe^II^ oxidation, however, occurred rapidly according to the Eh measured in samples from 2017, leading to the formation of poorly crystalline Fe oxyhydroxides such as ferrihydrite and lepidocrocite (Frohne et al., 2011; Yu et al., 2007), both of which increased in 2017 representing 40% of Fe minerals (Fig. 4). Contrarily long-term the contents of FeCR will mostly decrease since its formation is very low in environments with redox oscillations (Winkler et al., 2018). It should be noted, however, that poorly crystalline Fe minerals (i.e., FeLP and FeFR) phases are more susceptible to reduction within redox-oscillating environments (Nealson and Myers, 1992; Patrick and Jugsujinda, 1992; Reddy and DeLaune, 2008).

Thus, results showing the significant loss of Fe in the tailings-affected estuarine soils and the higher susceptibility of poorly crystallinity Fe oxyhydroxides to undergo reductive dissolution under transitory/cyclic anoxic conditions both point toward a decreasing capacity of poorly crystalline Fe minerals to control future Mn retention. The decrease in the significance of the Spearman correlation coefficient between total Fe and Mn in 2017 reinforces a less marked capacity of Fe oxyhydroxides in Mn retention (Fig. 9).

### 4.2. Environmental consequences

Metals can be absorbed into fish tissues through direct contact (i.e. through gills, skin or ingestion) such as with metals dissolved in the water column and associated with bottom sediments (Olsson et al., 1998; Weber et al., 2013). While the Cf in 2017 indicates low contamination levels of Mn in the soil at the studied site, high concentrations of Mn and other trace metals in the Rio Doce estuary suggested a high ecological risk for marine life (Bernardino et al., 2019; Gabriel et al., 2020a). Thus, the high contents of Mn in fish livers is indicative of chronic or acute exposure given the liver’s role in storage, redistribution, and metabolism of contaminants (Al-Yousuf et al., 2000). For these reasons, metal concentrations in the liver are useful indicators of bioaccumulation and are a bioindicator of Mn exposure in the Rio Doce estuary (Azevedo et al., 2009; Hauser-Davis et al., 2014). In fact, a recent study in the Rio Doce estuary indicated bioaccumulation of metals, including Mn, in tissues of *Cathoropus spixii* and *Genidens genidens* resulting in physiological effects due to chronic exposure to metal contaminants (Gabriel et al., 2020b).

The presence of Mn in fish muscle tissue poses a high risk to human health for the local community because the fish muscles are consumed by humans (Gabriel et al., 2020b; Gusso-Choueri et al., 2018). According to Gusso-Choueri et al. (2018), the consumption of metal-contaminated fish is one of the main routes of exposure for riverside communities. In most cases, the health risk is aggravated for those who depend on fish from the estuary to meet their daily dietary needs, which is the case for many people living near the Rio Doce estuary (Gabriel et al., 2020a).

According to the World Health Organization (WHO, 2011), food consumption is the primary route of exposure to Mn for humans, with the average concentration of Mn in staple protein sources such as beef, poultry, and fish ranging from 0.10 to 3.99 mg kg^−1^. Estimates for adequate daily consumption of Mn varies from 2 to 3 mg day^−1^ for adults (WHO, 2011). Therefore, according to the average Mn content found in fish muscles and the adequate daily consumption of Mn, the daily threshold consumption of *C. spixii* and *G. genidens* for an adult would be 2.5 kg and 5 kg, respectively, which may pose a real risk to the local population over the long-term due to presence of other sources of Mn exposure, such as drinking water, dust, fruit, vegetables, and dairy (Jolly et al., 2013; O’Neal and Zheng, 2015).

High concentrations of dissolved Mn measured in 2017 resulting from reducing conditions in the estuary is likely to have caused Mn accumulation in the tissues of the studied fishes (Arndt et al., 2014; Gabriel et al., 2020b; Pinsino et al., 2012). Our results from fish muscle and liver analyses show that Mn content in local fish species selected for this study are higher than Mn concentrations measured in other economically important fish species collected from areas that have also reported high concentrations of dissolved Mn (Table 2). Our findings suggest that fish living in the Rio Doce estuary may pose a chronic health risk for humans due to the elevated levels of tissue-bound Mn.

It is likely that the continued release of Mn from the estuarine soils will lead to Mn accumulation in other species of fish (Rather et al., 2019), crabs (Zhang et al., 2019), plants (Intawongse and Dean, 2006), and oysters (Silva et al., 2003), all of which are likely important food sources for the local population. The risks of Mn within the food chain are often overlooked in estuarine ecosystems because information on the toxic effect of Mn in aquatic organisms from these ecosystems is poorly studied despite recent studies that have suggested Mn induces oxidative stress, damage to tissues, inflammation and neurodegeneration in fish and crabs (Barrio-Parra et al., 2018; Vieira et al., 2012). Thus, this unnoticed toxicity of Mn increases the risk of bioaccumulation for the local population.

Moreover, a constant uptake of Mn trough food with high Mn concentrations a long-term may expose to local population to adverse human health effects promoted by high Mn accumulation as neurodegenerative disorder (Levy and Nassetta, 2003), cardiovascular toxicities (Jiang and Zheng, 2005), and liver damage (O’Neal and Zheng, 2015). In this sense, additional in vitro bio accessibility tests may be beneficial to provide toxicological issues that were not reported so far (Luo et al., 2012).

**Fig. 11.**
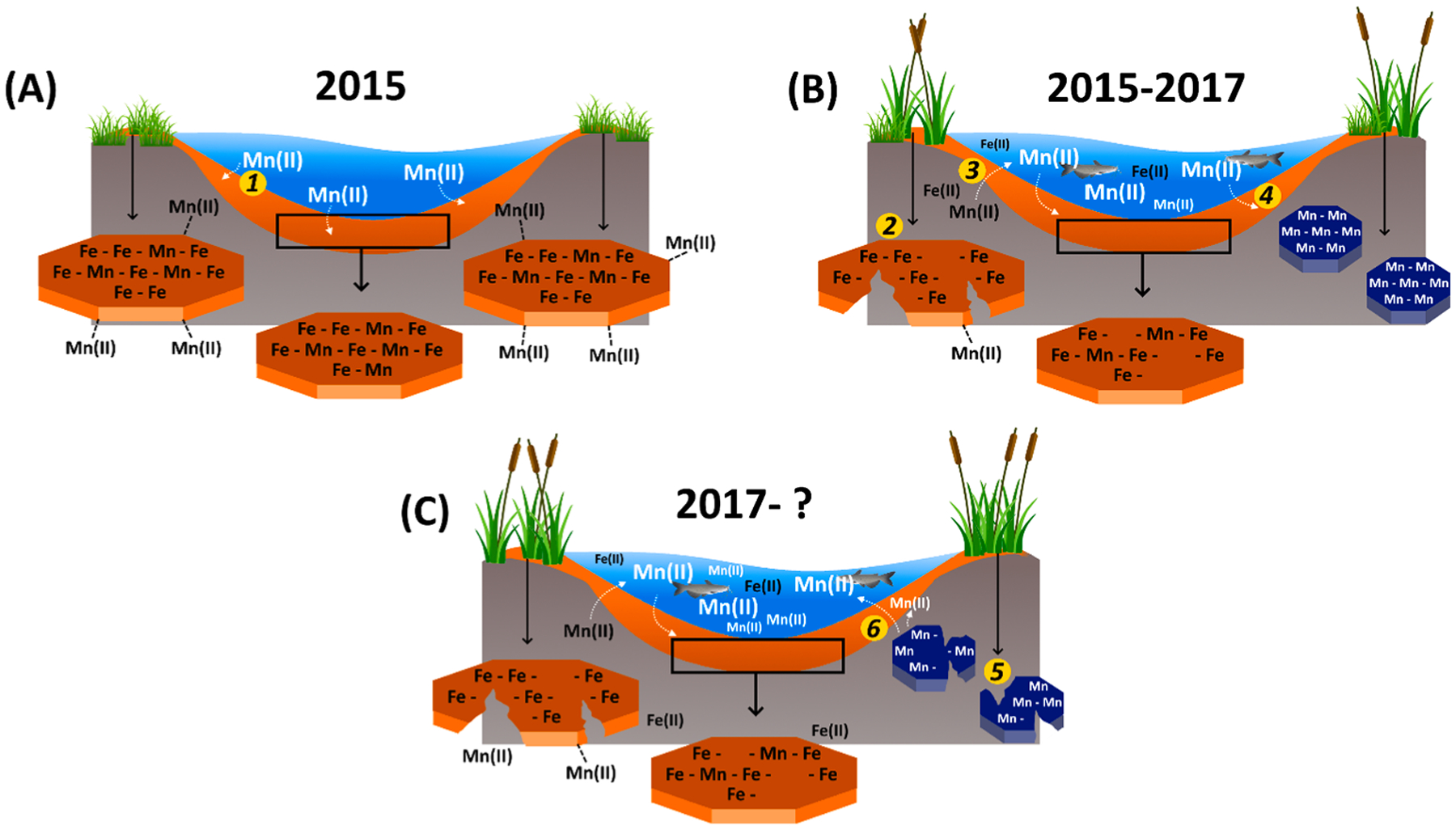
Schematic summary of the sequence of processes (order provided in yellow circles) leading to Fe and Mn mobilization in the Doce River estuary. The deposited mine tailings in 2015 from the Fundão dam rupture (Samarco mining company) led to an Fe oxyhydroxide and Mn enrichment in the estuary. Initially, Mn that arrived was predominantly immobilized on Fe oxyhydroxides (1). Between 2015 and 2017, plant growth promoted organic matter input to estuarine soils which stimulated the reduction of Fe oxyhydroxides (2) and subsequent release of dissolved Fe and Mn to estuarine waters, exposing fish and other wildlife to high concentrations of the metals (3). Redox oscillations caused by seasonal changes in precipitation and water levels, tidal flooding, plant activity, and fauna, has favored precipitation of poorly crystalline Fe oxyhydroxides and poorly crystalline Mn oxides (4). Mn oxides are easily reducible (5) which can contribute to an increase of Mn in estuarine water (6). Poorly crystalline Fe oxyhydroxides are also susceptible to reduction limiting their role in Mn retention in the future.

The continued downstream transport of the mine tailings accumulated along the Rio Doce basin will serve as a long-term source of Mn and other trace metals, and potentially maintain the continued bioaccumulation risks of Mn into the estuary. Therefore, chronic Mn contamination is expected to persist along with other trace metals, as a result of biogeochemical soil conditions that favor seasonal Fe and Mn oxide reduction, and the absence of other mineral fractions that can retain and immobilize Mn (except for poorly crystallinity Fe oxyhydroxides which exert ephemeral control; Fig. 11).

## 5. Conclusions

The Fe-rich mine tailings deposited from the Samarco disaster in the Rio Doce estuarine soils largely increased Mn concentrations in soil, water, and fish. The Fe minerals exert a temporary control on Mn bioavailability because crystalline Fe oxyhydroxides are gradually solubilized and replaced by poorly crystalline Fe oxides which can be easily reduced. Although the Mn released from Fe minerals may be reoxidized into poorly crystalline Mn oxides over time, the redox conditions in the Rio Doce estuary are highly conducive to reductive dissolution of Mn^III^ and Mn^IV^ containing poorly crystalline oxides, such as phyllomanganate and tectomanganates, which then ultimately leads to the continued increase in dissolved Mn.

Although Mn is considered an important micronutrient for all flora and fauna, the concentration of Mn found in the pore waters of the impacted estuary drastically exceeds the concentrations necessary for biological function leading to chronic Mn exposure. In this study, we found elevated Mn concentrations in liver and muscle tissues of multiple fish species that are regularly consumed by the local population. This discovery demonstrates that Mn sourced from the mine tailings can ultimately be impacting human health through long-term ecosystem contamination. Moreover, other animals and plants that are also consumed by the local population may accumulate Mn, worsening the risk to human health in the area.

## Supplementary Material

Supplementary

## Figures and Tables

**Table 1:** The relative abundance of solid-phase Mn^II^, Mn^III^, and Mn^IV^ within soil samples collected from multiple depths in 2017 and from the 0–3 cm depth in 2015 as determined by Mn K-edge XANES.

Sample	Mn K-edg e XANES			
	Mn^II^	Mn^III^	Mn^IV^	AMON
	Rel. abundance (%)*			
2015 (0–3 cm)	52	32	16	2.626
2017 (0–3 cm) – R1	34	33	33	2.992
2017 (0–3 cm) – R2	36	48	15	2.789
2017 (30–35 cm) – R1	55	24	21	2.668
2017 (30–35 cm) – R2	77	17	6	2.290

AMON = Average Mn oxidation number; R1: Replicate 1; R2: Replicate 2.

* Relative abundances determined using linear combination fitting.

**Table 2:** Mean values of Fe and Mn in liver and muscle for *Cathoropus spixii* and *Genidens genidens* collected 2017 from the Rio Doce estuary and for different commercial fish species worldwide.

Species	Total Fe		Total Mn		Reference
	Liver	Muscle	Liver	Muscle	
	mg kg^−1^				
*Cathoropus spixii*	*830 ± 638*	*26.75 ± 26.35*	*3.2 ± 1.78*	*1.0 ± 1.0*	*This study*
*Genidens genidens*	*1,541 ± 1,725*	*17.36 ± 11.4*	*2.1 ± 1.9*	*0.5 ± 0.2*	*This study*
*Silurus triostegus*	35.3 ± 6.6	n.d	n.d	n.d	Karadede et al. (2004)
*Lethrinus lentjan*	n.d	n.d	1.4 ± 0.2	0.1 ± 0.0	Al-Yousuf et al. (2000)
*Genidens barbus*	181 ± 100	3.87 ± 0.82	n.d	n.d	Avigliano et al. (2019)
*Chiloscyllium plagiosum*	n.d	n.d	0.2 ± 0.1	0.1 ± 0.0	Cornish et al. (2007)
*Mullus barbatus*	161.00 ± 25.30	29.20 ± 7.96	0.9 ± 0.2	0.4 ± 0.1	Tepe et al. (2008)
*Merlangius merlangus*	49.90 ± 7.16	21.90 ± 3.26	1.6 ± 0.2	0.4 ± 0.0	Tepe et al. (2008)
*Silurus glanis*	54.48 ± 17.59	10.17 ± 4.66	1.1 ± 1.4	0.5 ± 0.3	Andreji and Stráňai, (2007)

n.d: not determined.
