# Supplementary material for: Manganese: The overlooked contaminant in the world largest mine tailings dam collapse
Source: Environ Int. Author manuscript; Available in PMC 2021 Aug 24. (PMC8382573; doi:10.1016/j.envint.2020.106284)
Supplement: Supplementary [file NIHMS1732944-supplement-Supplementary.docx]

**Supplementary material**

Calibration, normalization and merging of replicate scans was done using the Demeter package (Ravel and Newville, 2005), version 9.26 with Larch running as a backend (Newville, 2013), and using a PC running Windows 10. The average Mn valence (AMV) of the Mn in each sample was obtained through linear combination fitting analysis of the Mn X-ray absorption near edge structure (XANES) spectra was done in Athena (Ravel and Newville, 2005) using the Combo method of Manceau et al. (2012). In all cases, the 17 reference spectra from Manceau et al. (2012) with the addition of two Mn^II^ standards (Mn-Oxalate and MnCl_2_) from the XAS standards repository in Demeter: (<https://github.com/bruceravel/demeter/tree/master/lib/Demeter/share/standards/data>) were used to perform unconstrained linear fits. Any reference yielding a negative loading was progressively removed on a per-sample basis and re-added to the reference list before fitting the next sample.

**Figure S1.** Manganese standards used in the linear combination fitting analysis of the Mn K-edge XANES. Standards obtained from Manceau et al. (2012) and from the Demeter XAS standard repository. * designates standards obtained from the Demeter XAS repository.


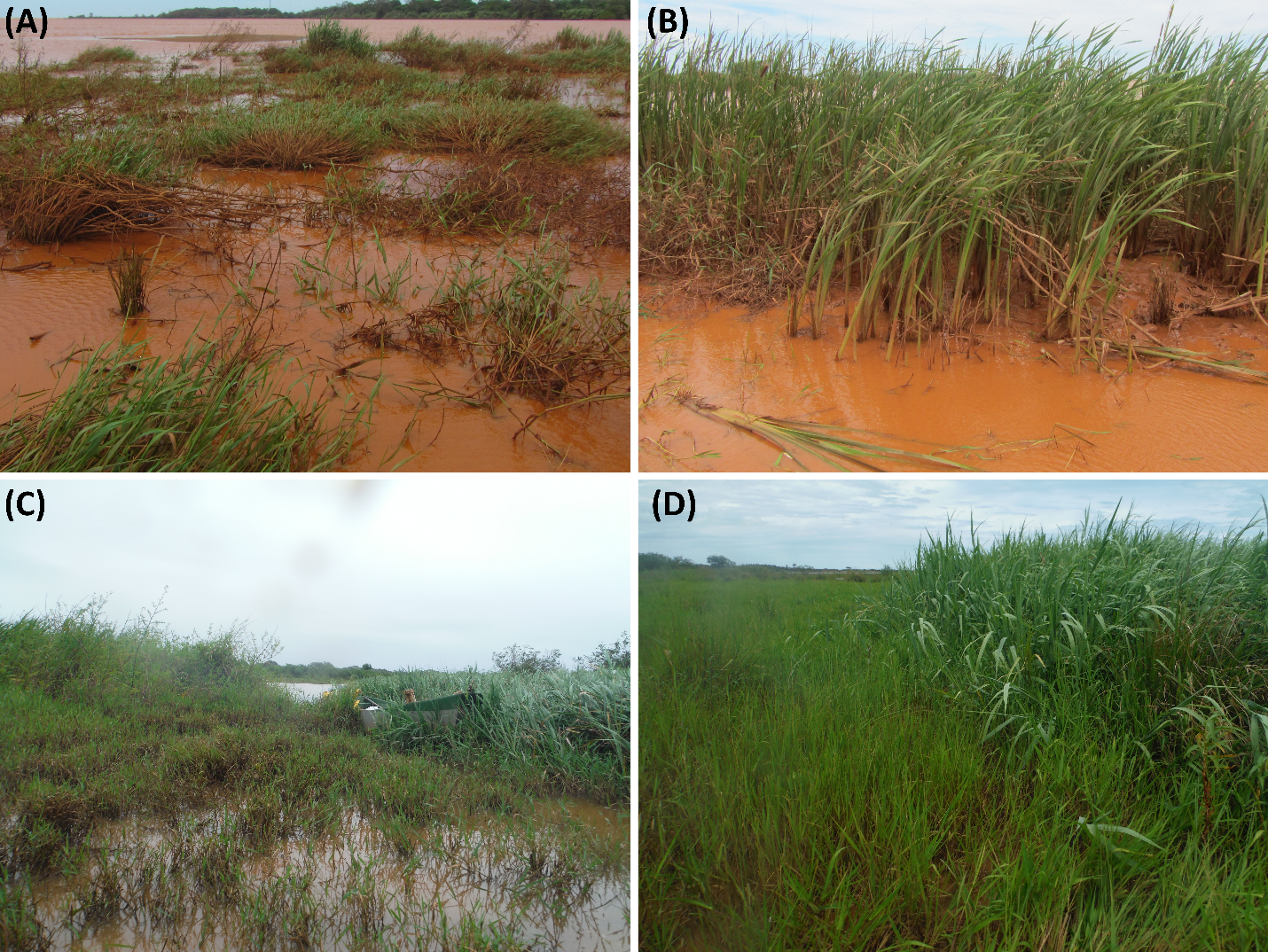


**Figure S2.** An overview of Rio Doce estuary in 2015 (A and B) showing the predominant vegetations in the Rio Doce estuary: *Typha domingensis* (A; courtesy of Xosé L. Otero), and *Eleocharis acutangula* (C; courtesy of Hermano M. Queiroz). In 2017 (C and D) an overview of the same areas showing the expansion of the surface occupied by macrophytes.


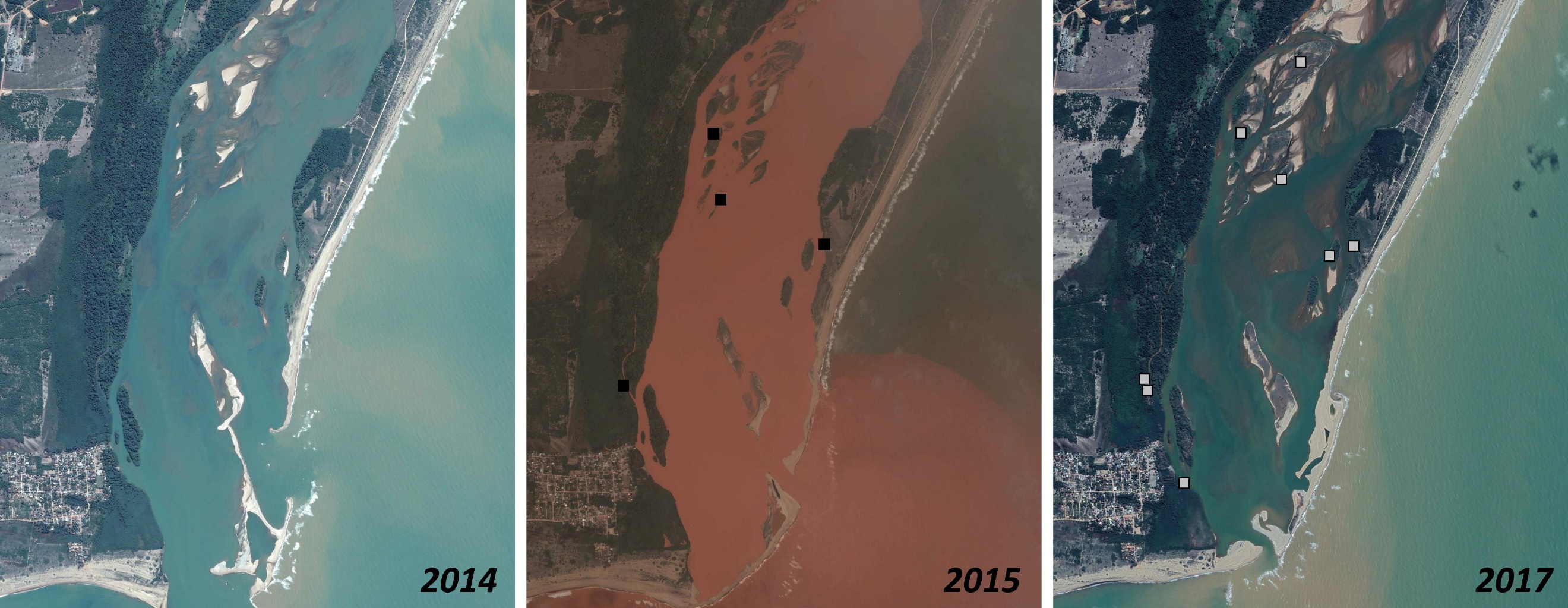


**Figure S3.** Location of soil sampling sites affected by tailings deposition in 2015 and 2017 in the Rio Doce Estuary, Regência, Espírito Santo, Brazil highlighting the permanent islands using satellite images from 2014, 2015, and 2017.

Table S1 – Description of solid-phase fractionation analysis of iron and manganese according to Tessier et al. (1979), Huerta-Diaz and Morse (1990), and Fortin et al. (1993).

| **Fraction** | **Abbreviation** | **Chemical Extractor/Procedure** |
| --- | --- | --- |
| Exchangeable and solube | EX | Extracted with a 1 mol L^-1^ MgCl_2_ solution at pH adjusted to 7 |
| Fe and Mn associated with carbonates | CA | Extracted with a 1 mol L^-1^ NaOAc (sodium acetate) solution at pH 5 |
| Fe and Mn associated with ferrihydrite and lepidocrocite, i.e, low crystallinity Fe phases | LC | Extracted with a 0.04 mol L^-1^ hydroxylamine + acetic acid 25 % (v/v) solution at 30 °C (ferrihydrite) and 96 °C (lepidocrocite) |
| Fe and Mn associated with hematite and goethite, i.e, high crystallinity Fe phases | CR | Extracted with a 0.25 mol L^-1^ sodium citrate + 0.11 mol L^-1^ sodium bicarbonate solution and 3 g of sodium dithionite at 75 °C |
| Fe and Mn associated to pyrite | PY | Extracted with concentrated HNO_3_. Before extraction the samples were subjected to treatment with 10 mol L^-1^ HF to remove phyllosilicates Fe, and concentrated H_2_SO_4_ was then added to remove Fe associated with organic matter |

Table S2 – Detection limits and quality assurance and quality control used in the ICP-OES for total content and iron fractionating analyses.

| **Quality assurance** | **Fe** | **Mn** |
| --- | --- | --- |
| Detection limit | 0.01 | 0.01 |
| Measured value | 9.026 | 9.769 |
| Certified value (NIST-1643f) | 10 | 10 |
| Recovery (%) | 90.3 | 97.7 |

NIST-1643F: Certified standard reference material for trace elements in water used on Mn and Fe determinations from extracts of total contents and iron fractionating analyses.

**References**

Fortín, D., Leppard, G.G., Tessier, A., 1993. Chactestis of lacustrine diagnetic iron oxyhydrodes 57, 4391–4404.

Huerta-Diaz, M.A., Morse, J.W., 1990. A quantitative method for determination of trace metal concentrations in sedimentary pyrite. Mar. Chem. 29, 119–144. https://doi.org/10.1016/0304-4203(90)90009-2

Manceau, A., Marcus, M.A., Grangeon, S., 2012. Determination of Mn valence states in mixed-valent manganates by XANES spectroscopy. Am. Mineral. 97, 816–827. https://doi.org/10.2138/am.2012.3903

Newville, M., 2013. Larch: An Analysis Package for XAFS and Related Spectroscopies. J. Phys. Conf. Ser. 430, 012007. https://doi.org/10.1088/1742-6596/430/1/012007

Ravel, B., Newville, M., 2005. ATHENA , ARTEMIS , HEPHAESTUS : data analysis for X-ray absorption spectroscopy using IFEFFIT. J. Synchrotron Radiat. 12, 537–541. https://doi.org/10.1107/S0909049505012719

Tessier, A., Campbell, P.G.C., Bisson, M., 1979. Sequential extraction procedure for the speciation of particulate trace metals. Anal. Chem. 51, 844–851. https://doi.org/10.1021/ac50043a017
